# Comparison of the Effectiveness of Pars Plana Vitrectomy with and without Internal Limiting Membrane Peeling for Idiopathic Retinal Membrane Removal: A Meta-Analysis

**DOI:** 10.1155/2015/974568

**Published:** 2015-11-26

**Authors:** Hanhan Liu, Shanru Zuo, Chun Ding, Xunzhang Dai, Xiaohua Zhu

**Affiliations:** ^1^Department of Ophthalmology, Second Xiangya Hospital, Central South University, Changsha 410011, China; ^2^The Third Xiangya Hospital of Central South University, China

## Abstract

We conducted a meta-analysis of published retrospective studies and compared the effectiveness of pars plana vitrectomy with and without internal limiting membrane (ILM) peeling for idiopathic epiretinal membrane (IERM). The results revealed that patients in the IERM+ILM peeling group had better BCVA after surgery within 12 months than those in IERM peeling group. But patients in the IERM peeling group showed better BCVA in the 18th month. More retrospective studies or randomized controlled trials are required to investigate and compare the long-term effect of IERM removal with and without ILM peeling.

## 1. Introduction

Idiopathic epiretinal membrane (IERM) is an avascular proliferative fibroblastic membrane with an unknown etiology that forms between the vitreous and internal limiting membrane. The prevalence of IERM reportedly ranges from 1.02% [1] to 18.5% [2] and it occurs more frequently in individuals under 50 years of age. Although the pathology of IERM remains unclear, its occurrence was found to be closely related to posterior vitreous detachment (PVD) or separation [3]. A number of cells, such as metaplastic retinal pigment epithelium (RPE) cells, glial cells, fibroblasts, and macrophages, are involved in the pathogenesis of IERM [4]. Furthermore, the dehiscent internal limiting membrane (ILM) formed during the development of PVD acts as a scaffold through which glial cells located posteriorly or hyalocytes located anteriorly migrate and proliferate on the retinal surface [5] resulting in the formation of a premacular membrane. IERM can remain transparent and asymptomatic for a long period of time; however, it can lead to blurred vision on opacification. Furthermore, IERM contraction can affect macular vision and cause metamorphopsia, micropsia, and monocular diplopia [6].

Pars plana vitrectomy with peeling of membrane has been used for treating symptomatic ERM for many years, although recurrence after successful surgery has been reported in 10% to 16.3% patients [7].

ILM is a homogeneous layer adhered to the posterior vitreous, formed by astrocytes and the end feet of Müller cells. It is separated from the vitreous humor by a basal lamina. Some surgeons believe that ILM peeling aids in the removal of residual IERM [8] and with increasing evidence showing the benefits of ILM peeling during IERM removal, including an improved visual acuity (VA) with a minimized recurrence rate [9] and superior retinal fold flattening [10], surgeons are increasingly using this procedure during IERM removal.

In contrast, some authors believe that ILM peeling may cause functional and mechanical damage to the Müller cells [11, 12]. Moreover, ILM peeling has been shown to result in a dissociated optic nerve fiber layer in the peeled area of the retina [13]. In addition, possible retinal toxicity caused by ILM staining is a concern that requires further investigation [11, 14, 15]. Meanwhile, several studies showed equivalent effectiveness and safety of IERM removal with and without ILM peeling [16–19]. Therefore, whether or not ILM peeling should be performed during vitrectomy for IERM removal remains controversial, and no comprehensive review has provided credible conclusions. Therefore, we conducted this meta-analysis of published retrospective studies to compare the effectiveness of pars plana vitrectomy with and without ILM peeling for IERM removal.

## 2. Materials and Methods

This meta-analysis was conducted according to the Preferred Reporting Items for Systematic Reviews and Meta-Analysis (PRISMA) guidelines [20]. No protocol exists for this systematic review.

### 2.1. Eligibility Criteria

The inclusion criteria were as follows: (1) comparative studies; (2) studies including patients with only idiopathic macular pucker, with IERM peeling performed in case and control groups; (3) studies with interventions including vitrectomy and including at least two groups (with and without ILM peeling); (4) studies with a minimum follow-up period of 3 months; (5) studies with at least two of the outcomes of interest, namely, pre- and postoperative best-corrected VA (BCVA) and vision improvement, recurrence rate, and complications; and (6) studies including patients aged over 18 years; there were no language restrictions; and (7) only studies with a MINOR score of >18 were included.

The exclusion criteria were as follows: (1) studies on secondary ERM resulting from retinal detachment, retinal vascular occlusion, uveitis, vitreous hemorrhage, trauma, or ocular tumors; (2) studies with inadequate data on pre- and postoperative BCVA; (3) studies including patients aged below 18 years; and (4) studies with subjects other than humans; and (5) studies with a MINOR score of ≤18 were excluded. The most detailed data were selected when sequential reports of the same cohort were identified.

### 2.2. Search Strategy

Databases including PubMed, the Cochrane library, EMBASE, Google Scholar, and the China National Knowledge Infrastructure (CNKI) were searched to retrieve related studies published before July 2015. “macular pucker” and “internal limiting membrane peeling” were used as sensitive terms along with “epimacular membrane,” “idiopathic macular epiretinal membrane,” “idiopathic epiretinal membrane,” “idiopathic macular,” “epiretinal membrane,” “preretinal macular fibrosis,” “epimacular proliferations,” “preretinal macular fibrosis,” “epiretinal fibrosis,” “epiretinal gliosis,” “surface wrinkling retinopathy,” and “cellophane maculopathy” as additional synonyms. The citations in the identified articles were then searched to retrieve additional studies. The reference lists of every primary article and previous systematic review were scrutinized for information about additional trials.

### 2.3. Study Selection and Data Collection

Two reviewers (using the Cochran's *Q* statistic and *I* ^2^ tests.) independently assessed studies on the basis of the title and abstract for possible eligibility. They then read the selected articles in detail and extracted the required data in a customized form. Any disagreement during data extraction was resolved by discussion. The author Stanley Chang was contacted for unpublished original data. The information extracted from each study included the first author, year, country, trial type, age, gender, preoperative BCVA, follow-up period, and recurrence rate. The outcomes of interest that were extracted included the following: postoperative BCVA; rate of increase in VA to ≥20/40; vision improvement, represented by VA improvement; recurrence rate; and postoperative complications, including retinal detachment, retinal tears, visual field defects, and macular edema.

### 2.4. Quality Assessment

The quality of the included studies was assessed using the Methodological Index for Nonrandomized Studies (MINORS) on a scale of 0 to 24 [21]. Studies with a score of ≥18 were considered to be of relatively high quality.

### 2.5. Statistical Analysis

The meta-analysis was conducted using the Review Manager Version 5.3 (Cochrane Collaboration, Oxford, United Kingdom) and Stata software (version 12.0; Stata Corp, College Station, Texas). Dichotomous outcomes were analyzed using pooled odds ratios (Ors). For continuous outcomes, analysis was performed using the weighted mean difference (WMD). Both Ors and WMDs were considered statistically significant at *P* < 0.05. Statistical heterogeneity among studies was evaluated using  |^2^ and *I* ^2^ tests. Both a fixed-effects model and a random-effects model were used to obtain summary Ors or WMDs. In the absence of heterogeneity between studies, the fixed-effects and random-effects model provided concordant results, and the random-effects model was employed only when heterogeneity was significant. The fixed-effects model was used to pool the data. Results obtained using the random-effects model are presented for cases of substantial heterogeneity. Potential publication bias was estimated by the Egger test. Publication bias was considered significant if the *P* value in Begg's test was <0.05.

## 3. Results

### 3.1. Study Selection

**Figure 1:**
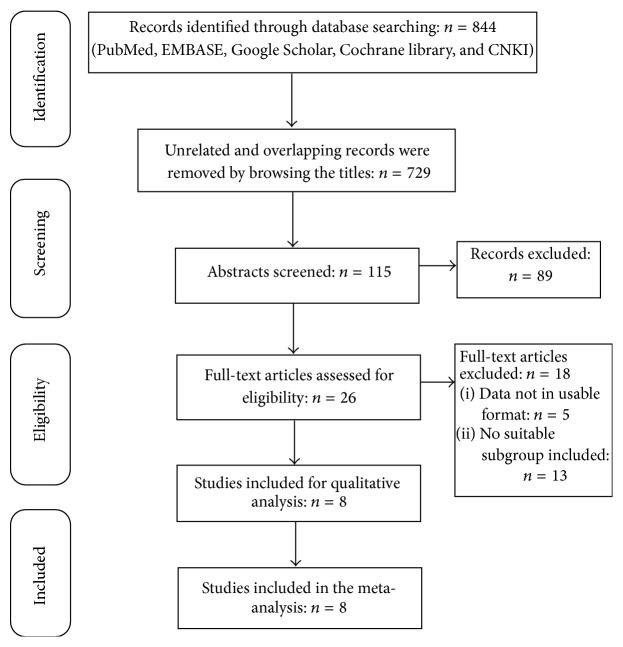
Study selection flowchart.

A total of 844 records were identified, 816 through database searching and 28 from article reference lists. By browsing the title and abstract, 818 unrelated and overlapped articles were removed. Twenty-six full-text articles were scrutinized for eligibility, and five without usable data and 13 that did not include suitable subgroups were excluded. Eventually, eight [15–19, 22–25] studies published from 2005 to 2015 were included in this meta-analysis.  Figure 1 provides a flow diagram of the study selection process.

### 3.2. Study Characteristics

*Quality Assessment and Characteristics of the Included Studies.* Table 1 shows the MINORS scores for the quality of included studies. In total, 418 eyes were included in this meta-analysis: 200 in the ILM peeling group (IERM+ILM peeling group) and 218 in the no ILM peeling group (IERM peeling group). All studies were retrospective with the following geographical distribution: six from Asia, one from Europe, and one from the USA. Two articles, one article each, were published in Chinese and Korean, while the remaining six were published in English. The detailed characteristics of the included studies and patients are shown in Table 2.  Table 3 shows the important details pertaining to the surgical methods, outcomes, and complications in these studies. The complications included punctate retinal hemorrhage, vitreous hemorrhage, cataract, and retinal detachment, with cataract being the most common.  Table 4 shows the postoperative BCVA values and vision improvement results, judged by an improvement in VA of ≥2 Snellen lines, while Table 5 shows the recurrence rates. ERM recurrence was defined as any evidence of a recurrent macular ERM on spectral domain optical coherence tomography (SD-OCT).

### 3.3. Efficacy Analysis

**Figure 2:**
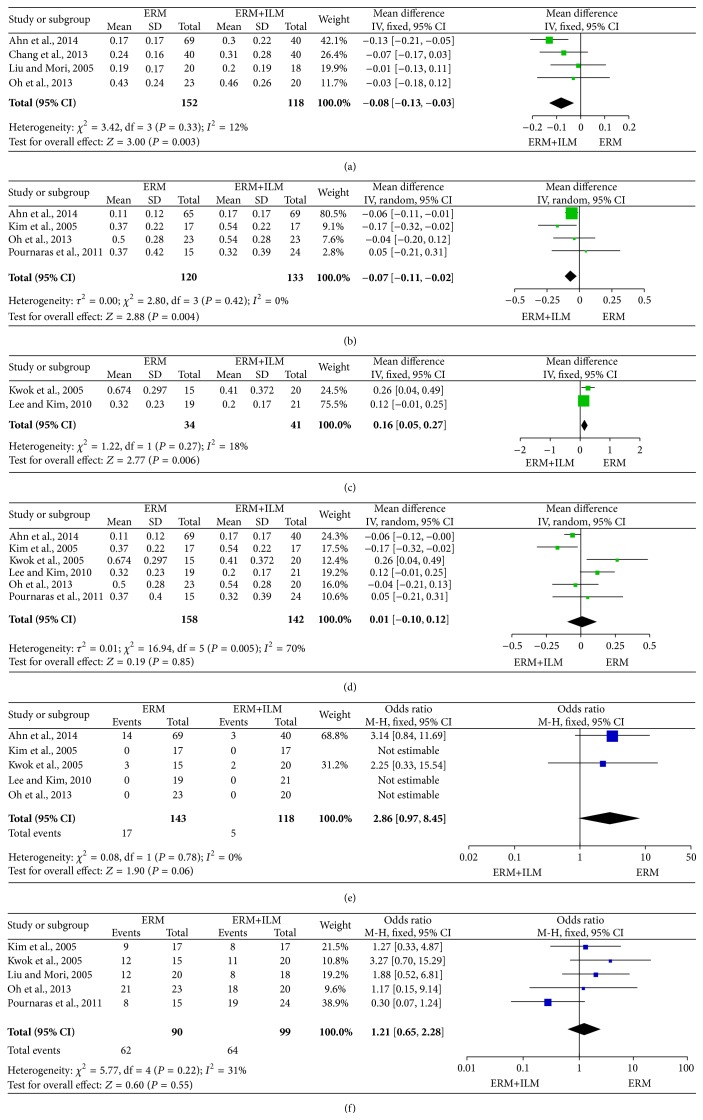
Visual outcomes of patients who underwent idiopathic retinal membrane peeling (IERM) only and those who underwent IERM + internal limiting membrane peeling. (a) Best-corrected visual acuity ≤6 months after surgery; (b) best-corrected visual acuity between 6 and 12 months after surgery; (c) best-corrected visual acuity in 18th month after surgery; (d) best-corrected visual acuity >6 months after surgery; (e) rate of improvement in visual acuity—by ≥2 Snellen lines; (f) recurrence rate.

The main results of the meta-analysis are presented in Figure 2. BCVA was analyzed ≤6 months, between 6 and 12 months, in the 18th month, and >6 months after surgery.  Figure 2(a) shows that BCVA ≤6 months after surgery was significantly better in the IERM+ILM peeling group than in the IERM peeling group (WMD = 0.08; 95% CI, −0.13 to −0.03, *P* = 0.003) as well as BCVA between 6 and 12 months (Figure 2(b); WMD = 0.07; 95% CI, −0.11 to −0.02, *P* = 0.004). But Figure 2(c) shows an opposite consequence in which BCVA in the IERM+ILM peeling group in the 18th month was worse than in the IERM peeling group (WMD = 0.16; 95% CI, 0.05 to 0.27, *P* = 0.006). Whereas after 6 months, no difference was observed between groups overall (Figure 2(d); WMD = 0.01; 95% CI, −0.10 to 0.12; *P* = 0.85). In addition, there were no differences in the rate of improvement in VA by ≥2 Snellen lines (Figure 2(e); OR = 1.21; 95% CI, 0.65 to 2.28; *P* = 0.55) and recurrence rates (Figure 2(f); OR = 2.86; 95% CI, 0.97 to 8.45; *P* = 0.06).

### 3.4. Heterogeneity, Sensitivity Analysis, and Publication Bias

Significant heterogeneity was observed in BCVA >6 months after surgery (*I* ^2^, 70%; *P* = 0.005). No publication bias was identified (Begg's test: *P* = 0.348 > 0.05, Egger's test *P* = 0.294 > 0.05). We conducted a metaregression analysis for BCVA according to study design, sample size, proportion of men, follow-up period at inclusion, and trial location. The results showed that the longest follow-up period of 18 months in the study by Lee and Kwok was the main source of heterogeneity (*P* = 0.024), explaining the differences between the primary studies. A sensitivity analysis conducted for the studies showed no significant differences (all CIs < 95%).

## 4. Discussion

This meta-analysis compared the effectiveness of pars plana vitrectomy with and without ILM peeling for IERM removal. The pooled results indicated that the postoperative BCVA was better within a short postoperative period (≤6 months and between 6 and 12 months) but worse in the 18th month in the IERM+ILM peeling group. In addition, BCVA after 6 months, rate of improvement in VA by ≥2 Snellen lines, and the recurrence rate were not significantly different between two groups. The complications varied among studies, with postoperative cataract being the most common.

The use of ILM peeling during pars plana vitrectomy for IERM removal remains controversial. The first report on macular ERM removal with ILM peeling indicated a less favorable visual outcome [26]. Park et al. [9] conducted a pilot study and suggested that ILM peeling during macular pucker surgery does not have deleterious effects and is associated with a low recurrence rate. Subsequently, various studies on ILM peeling during ERM removal were conducted to show diverse outcomes. However, till date, no meta-analysis evaluating the added benefits of ILM peeling during IERM removal has been conducted. Our meta-analysis is the first, as per our knowledge, to provide statistical results by comparing the benefits of pars plana vitrectomy with and without ILM peeling for IERM removal.

In some studies, significant correlation was observed between defect diameters of the cone outer segment tips (COST) line and BCVA after ERM removal [22, 27]. Inner segment (IS)/outer segment (OS) junction (Ellipsoid Zone) disruption was also found to contribute to poor VA among patients with ERM [28]. There was evidence showing mechanical damage of the photoreceptor layer [29] during ILM peeling. Also it is to be speculated that surgeons might remove not only the basement membrane of Müller cell membrane but also the end feet of Müller cells during ILM peeling, and this eliminates the contact of Müller cells with the nerve fibers [30, 31]. Therefore, ILM peeling might lead to substantial ultrastructural damage to the inner retinal surface, particularly in regions with a greater concentration of Müller cells, such as the regions between nerve fiber bundles [32]. But this could not explain why BCVA is worse in patients without ILM peeling during the first year after surgery. We speculate that peeling of ILM might help flatting retinal fold [10] and restoring normal photoreceptor structure.

In fact, Müller cell damage can manifest as b-wave abnormalities on multifocal macular electroretinography [33] or as concentric macular dark spots, a feature of the dissociated optic nerve fiber layer observed in the area of ILM peeling using en face SD-OCT 3 months after surgery [34]. In most cases, the ultrastructural changes are subclinical and do not seem to affect macular function as measured on the basis of visual acuity [18] although they can occasionally present clinically as microscotomas. The number of microscotomas was found to be significantly greater in the ILM peeling group than in the non-ILM peeling group in one study [35]. In a word, in addition to poor VA, microscotomas and decreased contrast sensitivity may cause daily discomfort for patients. Future studies should consider comparing the BCVA along with microscotomas numbers and vision-related quality of life before and after surgery [36].

In terms of vision improvement, in our meta-analysis, seven studies [16–19, 23–25] recorded favorable increases in visual acuity by ≥2 Snellen lines, although there were no significant differences between the IERM and IERM+ILM peeling groups. A previously published study reported that surgery for eyes with a preoperative vision of ≥0.25 has as much to gain as surgery for eyes with a preoperative vision of <0.25 [37]. We speculate that this may be due to irreversible microstructural damage before surgery in eyes with a preoperative vision of <0.25.

Staining was used in all included studies except one [24]. The difference in osmolarities of the indocyanine green (ICG) solutions used in the studies possibly contributed, in part, to the heterogeneity and may have also affected the visual outcome. It is widely accepted that stains harm the retinal cells [38, 39]. ICG staining induces lesions of the neurosensory retina and RP [39]. Although less toxic and safer dyes have been introduced [7], such as BBG and TA, no evidence shows that there are RNFL thickness differences related to the type of 3 vital stains.

In addition to the retrospective nature of the studies, differences in disease severity, and the relatively short follow-up periods, as an interval method, □ operation has its inherent weaknesses.

Firstly, surgical skill may play an essential role. Safe and accurate ILM peeling without staining is a difficult skill that requires a lot of practice. Furthermore, ILM and ERM may be clinically indistinguishable, and, without histopathological studies, we cannot exclude the possibility that some ILMs may have been peeled in some patients in the IERM peeling group. Approximately 40% to 100% of surgically removed ERM specimens demonstrate adherent ILMs [7, 26, 40, 41] and this rate can vary according to the skills of the surgeon and the condition of the patient. The rate of simultaneous ERM and ILM removal was 60% in the study of Oh et al. [16] and 81% in the study of Pournaras et al. [17]. This partly contributed to the similar outcomes between the two groups in these two studies and can create a problem in designing a random experiment because the possibility that some ILMs may have been peeled in some patients in the IERM peeling group cannot be excluded until surgery is complete.

In addition to unintended simultaneous ERM and ILM peeling, invisible residual tissue can also be present on the surface of the retina, subsequently affecting the postoperative VA [23]. Although experienced surgeons perform ERM removal with or without additional ILM peeling using almost identical procedures, one study showed that a larger ILM could be more easily peeled by senior surgeons [42]. Meanwhile, one study compared the forceps pinch-peel technique with the use of a diamond dusted membrane scraper for ILM peeling and indicated that the technique of ILM peeling can be another source of bias [43].

This study also has other limitations: first, the use of the Snellen chart for BCVA assessment has well-documented limitations such as inconsistent progression in letter size from one line to another and unequal legibility of the letters [44]; second, according to a previous study [7], the preoperative baseline BCVA and symptom durations correlated with the postoperative VA, because persistent and prolonged neuronal stretching and disruption can result in permanent damage that impacts VA and is irreversible even after the stretching ceases with a decrease in macular thickness after surgery [23]; third, although the study by Dugas et al. [45] showed equivalent functional and anatomical results for vitrectomy with ILM peeling and consecutive cataract surgery, the remarkable difference in the proportion of patients who underwent combined cataract surgery between the two groups may have affected the results of BCVA.

## 5. Conclusions

In conclusion, ILM peeling during IERM removal did not show better vision improvement or lower recurrence rate. Results also show that patients in the IERM+ILM peeling group had better BCVA after surgery within 12 months than those in IERM peeling group. But patients in the IERM peeling group showed better BCVA in the 18th month. Further prospective studies with a larger number of patients and longer follow-up periods are required to clarify the long-term effect of IERM removal with and without ILM peeling. Moreover, studies comparing the photoreceptor integrity between ERM removal with and without ILM peeling and between ILM peeling with and without the use of ICG staining should be performed.

## Figures and Tables

**Table 1:** Quality assessment using the Methodological Index for Nonrandomized Studies.

Study	1	2	3	4	5	6	7	8	9	10	11	12	MINORS score

Kim et al., 2005 [19]	2	2	2	2	2	2	2	2	1	2	0	2	21
Kwok et al., 2005 [25]	2	1	2	2	2	2	2	2	0	2	2	2	21
Liu and Mori., 2005 [24]	2	2	2	2	2	2	2	2	1	2	0	2	21
Lee and Kim, 2010 [18]	2	2	2	2	2	2	2	2	1	2	0	2	21
Pournaras et al., 2011 [17]	2	2	2	2	2	2	2	2	0	2	0	2	20
Chang et al., 2013 [23]	2	2	2	2	2	1	2	2	1	0	2	2	20
Oh et al., 2013 [16]	2	2	2	2	2	2	2	2	0	2	0	2	20
Ahn et al., 2014 [22]	2	2	2	2	2	2	2	2	1	2	0	2	21

(1) A stated aim of the study; (2) inclusion of consecutive patients; (3) prospective collection of data; (4) endpoints appropriate for the study aim; (5) unbiased evaluation of endpoints; (6) follow-up period appropriate to the major endpoint; (7) loss to follow-up not exceeding 5%; (8) a control group having the gold standard intervention; (9) contemporary groups; (10) baseline equivalence of groups; (11) prospective calculation of the sample size; (12) statistical analyses adapted to the study design.

0: not reported; 1: reported but inadequate; 2: reported and adequate.

**Table 2:** Characteristics of the included trials.

Study	Country	Study design	Pts/eyes (*n*)		Mean age (year)		Gender (M/F)		Follow-up (mo)		Preoperative BCVA	
			E	E+I	E	E+I	E	E+I	E	E+I	E	E+I
Kim et al., 2005 [19]	Korea	Retrospective comparative	17	17	61.6 (43~78)	63.5 (48~79)	7/10	12/5	11.2 (6~27)	8.9 (6~11)	0.71 ± 0.50	0.71 ± 0.16
Kwok et al., 2005 [25]	Hong Kong	Retrospective	15	20	67.9 ± 8.0	63.9 ± 9.3	13/2	2/18	50.2 ± 18.0	23.6 ± 5.4	0.90 ± 0.293	0.652 ± 0.429
Liu and Mori, 2005 [24]	Japan	Retrospective	20	18	68.2 ± 6.9	69.4 ± 5.7	9/11	9/9	>3 months		0.35 ± 0.26	0.44 ± 0.21
Lee and Kim, 2010 [18]	Korea	Retrospective	19	21	65.47 ± 7.66	63.43 ± 7.18	3/16	4/17	18.32 ± 12.03	18.05 ± 11.81	0.67 ± 0.34	0.68 ± 0.21
Pournaras et al., 2011 [17]	Switzerland	Retrospective	15	24	77.1 ± 6.7	73.3 ± 10.6	7/8	13/11	41.9 ± 35.6	24.0 ± 12.6	0.48 ± 0.22	0.58 ± 0.40
Chang et al., 2013 [23]	America	Retrospective	40	40	70.5	70.1	—	—	3 months		0.44 ± 0.18	0.52 ± 0.31
Oh et al., 2013 [16]	Korea	Retrospective	23/23	20/20	64.0	65.3	10/13	7/13	>12 months		0.35 ± 0.16	0.44 ± 0.21
Ahn et al., 2014 [22]	Korea	Retrospective	65/69	37/40	63.9 ± 11.1	64.3 ± 10.0	32/37	18/22	>6 months		0.31 ± 0.21	0.38 ± 0.19

E: epiretinal membrane peeling only.

E+I: epiretinal membrane + internal limiting membrane peeling.

**Table 3:** Surgery-related features of the included trials.

Study	Surgeon	Vitrectomy	Dying	Peeling diameter	Pseudophakic		Outcomes	Complications
					E	E+I		
Kim et al., 2005 [19]	—	—	0.5% ICG	2 PD	Pre: 5	Pre: 2	I III IV	Cataract^*∗*^9
					Post: 13	Post: 12		

Kwok et al., 2005 [25]	1	20 G	1 mg/mL ICG	3-4 PD	Some		I II III	Retinal detachment^*∗*^1

Liu and Mori, 2005 [24]	—	—	—	—	Except 1 in E group		I II	—

Lee and Kim, 2010 [18]	1	—	0.125% ICG	—	16	18	I II IV	0

Pournaras et al., 2011 [17]	1	20/23 G	0.15% ICG	—	All		I II IV	0

Chang et al., 2013 [23]	1	20 G	BBG	—	Some		I II	—

Oh et al., 2013 [16]	1	20 G	0.5% ICG	2 PD	Pre: 5 Post: 6	Pre: 4 Post: 5	I II	Cataract^*∗*^11 Vitreous hemorrhage^*∗*^1 Punctate retinal hemorrhage^*∗*^27

Ahn et al., 2014 [22]	2	23 G	0.05% ICG	—	Pre: 10		I III	0
					Post: 36	Post: 28		

I: Pre- and postoperative best-corrected visual acuity; II: vision improvement; III: recurrence rate; IV: complications.

**Table 4:** Postoperative best-corrected visual acuity (logMAR) and vision improvement (VI).

Study	Postoperative BCVA and visual improvement			
	IERM peeling	IERM+ILM peeling	*P* value (1)	*P* value (2)
Kim et al., 2005 [19]	0.37 ± 0.22 (>6 mo)	0.54 ± 0.22 (>6 mo)	0.413/0.18	0.012/0.031
	VI: 9/17	VI: 8/17	>0.05	

Kwok et al., 2005 [25]	0.674 ± 0.297 (18 mo)	0.41 ± 0.372 (18 mo)	—	—
	VI: 12/15	VI: 11/20	—	

Liu and Mori, 2005 [24]	0.19 ± 0.17 (3 mo)	0.20 ± 0.19 (3 mo)	>0.05/>0.05	<0.01/<0.05
	VI: 12/20	VI: 8/18	—	

Lee and Kim, 2010 [18]	0.32 ± 0.23 (18 mo)	0.20 ± 0.17 (18 mo)	0.784/0.11	0.001/0.000
	VI: 0.36 ± 0.30	VI: 0.48 ± 0.16	0.095	

Pournaras et al., 2011 [17]	0.37 ± 0.42 (>6 mo)	0.32 ± 0.39 (>6 mo)	>0.1/>0.1	—
	VI: 8/15	VI: 19/24	—	

Chang et al., 2013 [23]	0.24 ± 0.16 (3 mo)	0.31 ± 0.28 (3 mo)	0.15/0.13	—
	VI: 0.2 (avg.)	VI: 0.21 (avg.)	0.88	

Oh et al., 2013 [16]	0.40 ± 0.18 (3 mo)	0.56 ± 0.26 (3 mo)	0.157/0.027	0.095/0.009
	0.43 ± 0.24 (6 mo)	0.46 ± 0.26 (6 mo)	0.667	0.026/0.171
	0.50 ± 0.28 (12 mo)	0.54 ± 0.28 (12 mo)	0.74	0.011/0.017
	VI: 21/23	VI: 18/20	0.740	

Ahn et al., 2014 [22]	0.17 ± 0.17 (1 mo)	0.30 ± 0.22 (1 mo)	0.12/0.001	<0.001/<0.001
	0.11 ± 0.12 (12 mo)	0.17 ± 0.17 (12 mo)	0.15	
	VI: —	VI: —	—	

(1) Difference between preoperative/postoperative IERM peeling and IERM+ILM peeling group.

(2) Difference between preoperative and postoperative IERM peeling group/preoperative and postoperative IERM+ILM peeling group.

**Table 5:** Recurrence rate.

Study	Recurrence rate		
	IERM peeling	IERM+ILM peeling	*P*
Kim et al., 2005 [19]^*∗*^	0	0	—
Kwok et al., 2005 [25]^*∗*^	3/15	2/20	—
Liu and Mori, 2005 [24]	—	—	—
Lee and Kim, 2010 [18]^*∗*^	0	0	—
Pournaras et al., 2011 [17]	—	—	—
Chang et al., 2013 [23]	—	—	—
Oh et al., 2013 [16]^*∗*^	0	0	—
Ahn et al., 2014 [22]^*∗*^	14/69	3/40	0.06

IERM: idiopathic epiretinal membrane; ILM: internal limiting membrane.

^*∗*^The timespan of recurrence rate: Kim et al., 2005 [19]: 6–27 months; Kwok et al., 2005 [25]: 18 months; Oh et al., 2013 [16]: 12 months; Lee and Kim, 2010 [18]: 5–44 months; Ahn et al., 2014 [22]: 12 months.
